# Diffuse neuroinflammation and immature neuron loss in fetal Rhesus macaques after short-term intrauterine infection

**DOI:** 10.1186/s12974-025-03686-y

**Published:** 2026-01-15

**Authors:** Pietro Presicce, Danielle Beckman, Giovanne B. Diniz, Monica Cappelletti, Sean Ott, Sivan Bercovici, Shiv Kale, Paul Babb, Jyodi Mohole, Lauren S. Richardson, Ananth K. Kammala, Ramkumar Menon, Lisa A. Miller, Elizabeth E. Crouch, Alan H. Jobe, Senad Divanovic, Claire A. Chougnet, Sing Sing Way, John H. Morrison, Suhas G. Kallapur

**Affiliations:** 1 Divisions of Neonatology and Developmental Biology, David Geffen School of Medicine at UCLA, Mattel Children’s Hospital UCLA, 10833 Le Conte Avenue, Room B2-375 MDCC, Los Angeles, CA 90095 USA; 2 California National Primate Research Center, University of California Davis, Davis, CA USA; 3 Karius, Inc, Redwood City, CA USA; 4 Division of Basic Science and Translational Research, Department of Obstetrics and Gynecology, The University of Texas Medical Branch at Galveston, Galveston, TX USA; 5 Department of Anatomy, Physiology, and Cell Biology, School of Veterinary Medicine, University of California Davis, Davis, CA USA; 6 Department of Pediatrics, University of California San Francisco, San Francisco, CA USA; 7 Division of Neonatology/Pulmonary Biology, Cincinnati Children’s Hospital Research Foundation and the University of Cincinnati College of Medicine, Cincinnati, OH USA; 8 Division of Immunobiology, Cincinnati Children’s Hospital Research Foundation and the University of Cincinnati College of Medicine, Cincinnati, OH USA; 9 Division of Infectious Diseases, Cincinnati Children’s Hospital Research Foundation and the University of Cincinnati College of Medicine, Cincinnati, OH USA

**Keywords:** Chorioamnionitis, Cerebral palsy, Neuroinflammation, Prematurity, Neurobehavior disorder, Autism spectrum defect

## Abstract

Chorioamnionitis or infection of the amniotic fluid and membranes surrounding the fetus can cause fetal systemic inflammation and adverse neurological outcomes. To investigate the pathogenesis of chorioamnionitis-induced neurological injury, we developed a non-human primate model by infusion of live *Escherichia coli*. A micro-osmotic pump was placed in pregnant Rhesus macaques at ~ 85% term gestation via hysterotomy. Live *E. coli* (10^5^ CFU/h, 1µL/h, *n* = 7) or sterile broth (Controls, 1µL/h, *n* = 7) infusions were given over 72 h followed by necropsy. A third group of pregnant macaques received microbicidal antibiotics 24 h after the start of *E. coli* infusion (*n* = 5). Amniotic fluid, cord plasma, and fetal brain regions were carefully preserved for comparative analysis of fetal systemic and brain inflammation. *E. coli* exposure caused fetal systemic inflammation without bacteremia and induced pro-inflammatory cytokines in the periventricular white matter (PVWM) and thalamus. A diffuse inflammation characterized by aberrant proliferation and activation of IBA1^+^ microglia and GFAP^+^ astrocytes was observed in multiple brain regions. There was a selective vulnerability of the DCX expressing immature neurons with relative sparing of cortical mature neurons expressing MAP2 or NeuN. Periventricular and entorhinal white matter regions were particularly vulnerable with relative sparing of the cortex. These pathogenic changes of fetal brain injury were observed after 72 h exposure. Maternal antibiotics cleared the bacteremia but did not decrease neuroinflammation or neuropathology. These results highlight the potential need for anti-inflammatory adjunctive therapy in addition to antibiotics for optimal outcomes in chorioamnionitis.

**Supplementary Information:**

The online version contains supplementary material available at 10.1186/s12974-025-03686-y.

## Introduction

Chorioamnionitis is characterized by infection or inflammation in the fetal membranes surrounding the fetus or the amniotic fluid. Although preterm infants are more commonly exposed to chorioamnionitis with rates ~ 70% at 24 weeks gestation [1, 2], 5–12% of term pregnancies are also affected [3]. Epidemiological data for the association between exposure to chorioamnionitis and adverse neurological outcomes during childhood is exceptionally strong with risk ratios of the exposed group 1.4 to ninefold higher than non-exposed for different neurological disorders [4–7]. As can be expected, prematurity further increases the risk for chorioamnionitis-induced adverse neurological outcomes in childhood [6, 8–10], although it is worth highlighting that both term and preterm infants are susceptible to chorioamnionitis induced brain injury.

A well-documented consequence of chorioamnionitis is the fetal inflammatory response syndrome (FIRS). FIRS is characterized by increases in cord blood cytokines such as IL6 and acute phase reactant proteins such as C-reactive protein [11, 12]. We now understand that FIRS is surprisingly common, affecting ~ 3% of all births in the US [13]. Importantly, FIRS is also associated with later onset of autism spectrum defects (ASD) [14–16] and cerebral palsy [4]. Although lower genital microorganisms cause chorioamnionitis [17–20], most of the infants with FIRS do not have demonstrable microorganisms in the blood [21]. Furthermore, maternal antibiotic treatment is ineffective in preventing chorioamnionitis/FIRS induced adverse neurological outcomes [16, 22]. Additional findings suggest that, in some cases, maternal antibiotics may even exacerbate the risk for ASD [23].

Information regarding precise brain regions or cell types that are injured by chorioamnionitis/FIRS remain a significant knowledge gap. Understanding regional and cellular differences in vulnerability to chorioamnionitis/FIRS in the fetal brain is particularly important given the dynamic maturation of neuronal cell types and cortical connections during development. Of note, some regions of the brain such as the subplate zone are uniquely found in the developing fetal brain [24].

Given the distinctly similar gestational anatomy, reproductive immunology, we chose to develop a non-human primate (NHP) model at ~ 85% term gestation. Although fetal brain development trajectory between human and Rhesus macaque fetuses are largely parallel [25], neuro-imaging studies reveal that Rhesus macaque brain at 85% gestation is more similar to 42-week gestation human brain (post-term) [26]. Chorioamnionitis was induced by infusion of live *E. coli* in the choro-decidua (CD) space of the fetal membranes. Additionally, to understand if antibiotics protect against neuropathology, we gave microbicidal antibiotics to the mother starting 24 h after bacterial infusion. Our data show that CD *E. coli* infusion causes FIRS without bacteremia resulting in a microglia/astroglia-driven neuro-inflammation. Periventricular and entorhinal white matter regions were particularly vulnerable with relative sparing of the cortex. Among neuronal cell types, immature neurons expressing DCX were more vulnerable to injury compared with mature neurons expressing MAP2 or NeuN. Importantly, maternal antibiotics did not protect fetuses from neuro-inflammation. Our data thus provide important mechanistic insights into how chorioamnionitis/FIRS causes adverse neurological outcomes.

## Results

### Chorio-decidua (CD) infusion of live *E. coli* causes localized infection and fetal inflammation without bacteremia

**Fig. 1:**
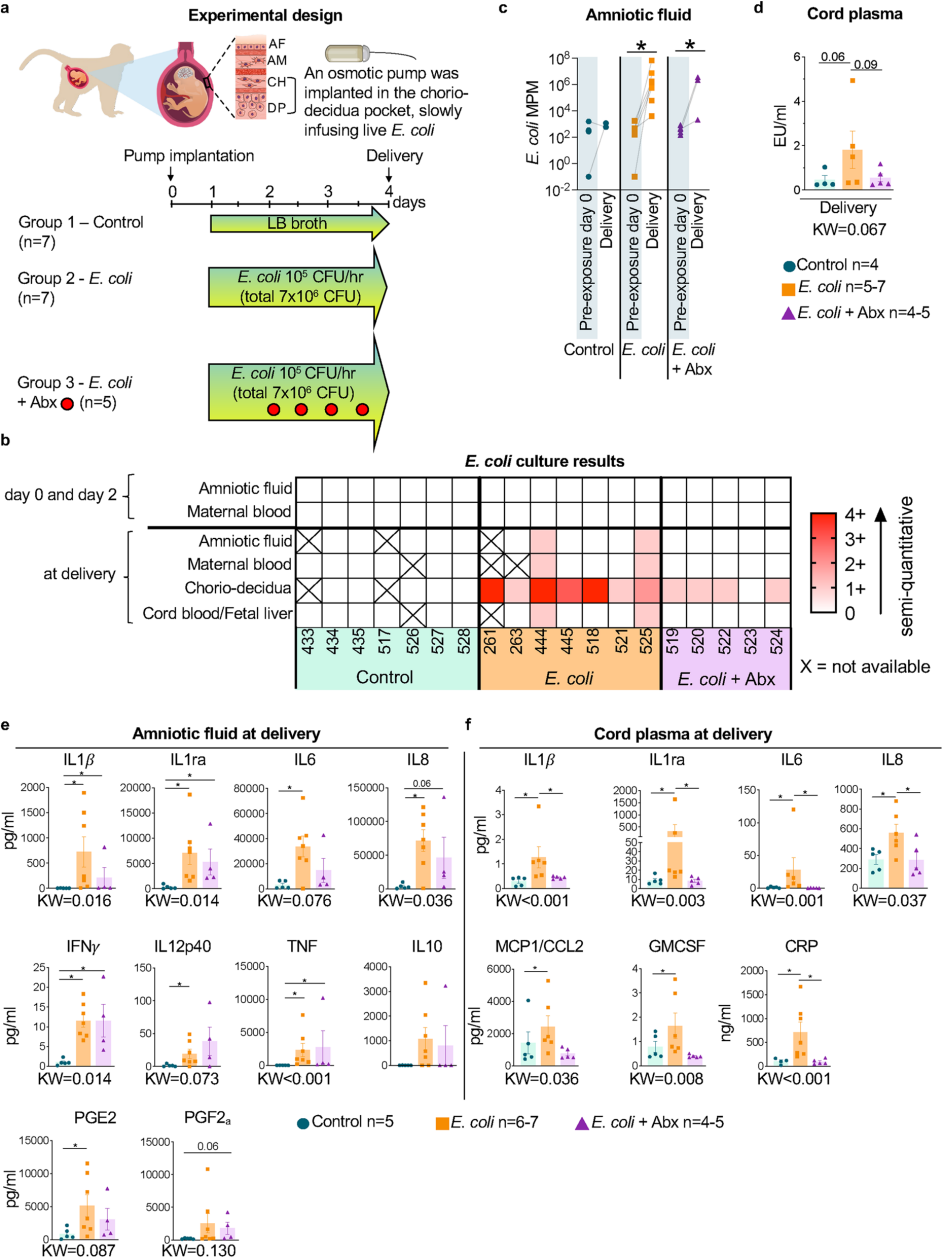
Infusion of live *E. coli* in the chorio-decidua induces localized infection and fetal inflammation without bacteremia.** a** Experimental model of micro-osmotic pump implantation and infusion regimen for the three groups of animals (see methods for details). AF = amniotic fluid; AM = amnion; CH = chorion; DP = decidua parietalis.** b** *E. coli* invasion in different fluids/tissues and time points (day 0 = day of pump implantation, day 1 is start of *E. coli*/broth infusion) is shown as a heat map depicting semi-quantitative growth using traditional culture methods. The numbers shown for each column represent unique animal IDs. Note 1 + to 4 + *E. coli* growth (on a scale of 0 to 4 +) at delivery in CD of *E. coli*-exposed animals (Group 2) and a 0 to 1 + growth in the CD of Group 3 animals exposed to antibiotics (Abx). Only 2/7 animals of Group 2 showed a 1 + *E. coli* growth in the amniotic fluid, maternal, and umbilical cord blood at delivery (these animals had no growth in AF and maternal blood on day 2). **c** *E. coli* cell free DNA depicted as molecules per microliter (MPM) significantly increased in the amniotic fluid of Group 2 and Group 3 but not in Group 1 animals between day 0 and delivery. **d** Umbilical cord plasma endotoxin activity depicted as endotoxin units (EU) per mL increased (*p* = 0.06) in Group 2 compared to Group 1 animals. Antibiotics decreased endotoxin activity (Group 3 vs Group 2 *p* = 0.09). Pro-inflammatory markers were measured in **e** AF and** f** Umbilical cord plasma at delivery by multiplex ELISA**. e** AF IL1β, IL1ra, IL6, IL8, IFNγ, IL12p40, TNF alpha, and PGE2 significantly increased upon *E. coli* exposure. Abx treatment did not decrease these cytokines. **f** Cord plasma IL1β, IL1ra, IL6, IL8, MCP1/CCL2, GM-CSF, and C-reactive protein (CRP) significantly increased upon *E. coli* exposure compared to saline controls. Abx treatment significantly decreased IL1β, IL1ra, IL6, IL8, and CRP. For panel **c**, paired t test was used to compare cfDNA at day 0 vs delivery within the same subject. For other panels data are mean ± SEM. Initial comparison between three groups were done using Kruskal–Wallis (KW) non-parametric test with p value shown at the bottom of each graph. For two group comparisons, non-parametric Mann–Whitney U test was used (*p < 0.05 between comparators; the actual p values between 0.05 and 0.1 is shown)

To model the gradual ascension of microorganisms from the maternal lower genital tract to the CD space, we implanted a micro-osmotic pump to infuse live *E. coli* into the CD pocket of pregnant Rhesus macaques at ~ 85% gestation (Fig. 1a, see methods for details). *E. coli* was chosen as a model organism to induce chorioamnionitis/FIRS as it is the most common bacteria isolated in early onset neonatal sepsis resulting from chorioamnionitis in the preterm [27]. Three groups of animals were studied: 1) Controls *n* = 7, 2) Live *E. coli* *n* = 7 (*E. coli*), 3) Live *E. coli* + delayed start of maternal antibiotics (Abx) *n* = 5. All animals had surgery to place a CD micro-osmotic pump. Group 1 had sterile LB broth infusion, while groups 2 and 3 had live *E. coli* infusion 7 × 10^6^ CFU over 72 h. There were no maternal/fetal deaths. A subset of dams (Group 1: 3/7, Group 2: 1/7, Group 3: 2/5) delivered during the 72 h infusion due to preterm labor likely caused by surgical manipulation of the uterus (Suppl. Table 1). None of the animals received tocolytic therapy. The duration of *E. coli*/broth exposure was 72 h except for those delivering prematurely. In the premature delivery subset, average duration of exposure was 48 h for controls, 48 h for *E. coli* only and 66 h for *E. coli* + Abx groups. All animals regardless of preterm labor were included in this study of a limited sample size since the brain and FIRS measurements had a similar distribution within each group.

The infusion of live *E. coli* without added antibiotics caused a robust localized infection with purulent exudate, characterized by a massive infiltration of neutrophils at the chorio-decidua junction (Suppl. Figure 1). Consistently, culture of the CD pocket at delivery was strongly positive (median 3 +, on a scale from 0 to 4 +) for *E. coli* growth (Fig. 1b). Amniotic fluid (AF) and maternal blood cultures were negative at baseline (day 0) and on day 2 (Fig. 1b). However, at delivery, 2/6 subjects that were previously negative had a faint (1 +) *E. coli* growth in the AF and maternal blood (Fig. 1b). As expected, none of the sham infection surgical control animals had *E. coli* growth in CD/body fluids at any time point (Fig. 1b). Maternal antibiotic therapy was effective in reducing CD *E. coli* growth from heavy to mild growth (3 +/4 + to 1 +) at delivery, with 1/5 subjects reporting no growth (Fig. 1b). Notably, none of the animals in the Abx group had *E. coli* growth in the AF or maternal blood at any time point (Fig. 1b). Next, we measured cell-free *E. coli* DNA since bacterial DNA is a potent activator of host immune response and found in circulation long after bacterial killing by antibiotics [28, 29]. Despite no growth, *E. coli* cell free DNA copy number increased significantly several log-orders from d0 to delivery in the AF only in *E. coli* exposed groups but not in controls. Interestingly, Abx did not affect the longitudinal increase in *E. coli* cell-free DNA within the same subject, (Fig. 1c). Cord blood microbial cell free DNA could not be measured as plasma collected in heparin anticoagulant was not compatible with the assay platform. Although cord blood cultures showed no growth, there was a fourfold increase in endotoxin activity in the cord blood of *E. coli* exposed fetuses (p = 0.06) compared to controls (Fig. 1d).

To examine the functional impact of CD *E. coli* infection, we measured inflammatory mediators. *E. coli* increased significantly AF levels of pro-inflammatory cytokines IL1β, IL6, IL8, IFNγ, IL12p40, TNF, and prostaglandin PGE2, but not prostaglandin PGF2_a_ (Fig. 1e). The increases were large (highest for IL6 and IL8) compared to very low levels in the control animals who had the same surgical procedures as the experimental animals (Fig. 1e). Anti-inflammatory cytokine IL1ra increased but IL10 did not significantly increase after *E. coli* exposure (Fig. 1e). Abx did not decrease significantly *E. coli* induced AF cytokines.

In the cord blood, *E. coli* exposure increased concentrations of pro-inflammatory cytokines IL1β, IL6, IL8, CCL2, GMCSF (CSFII), C-reactive protein (CRP), and the anti-inflammatory cytokine IL1ra (Fig. 1f). Overall, cord blood cytokine levels were 3 log-orders lower compared to the AF from the same animal. Antibiotics decreased the levels of most *E. coli* induced cord blood cytokines and that of CRP, however CCL2, GMCSF levels (Fig. 1f) and endotoxin activity (Fig. 1d) were not statistically different.

### *E. coli* increased levels of pro-inflammatory cytokines in the fetal brain

**Fig. 2:**
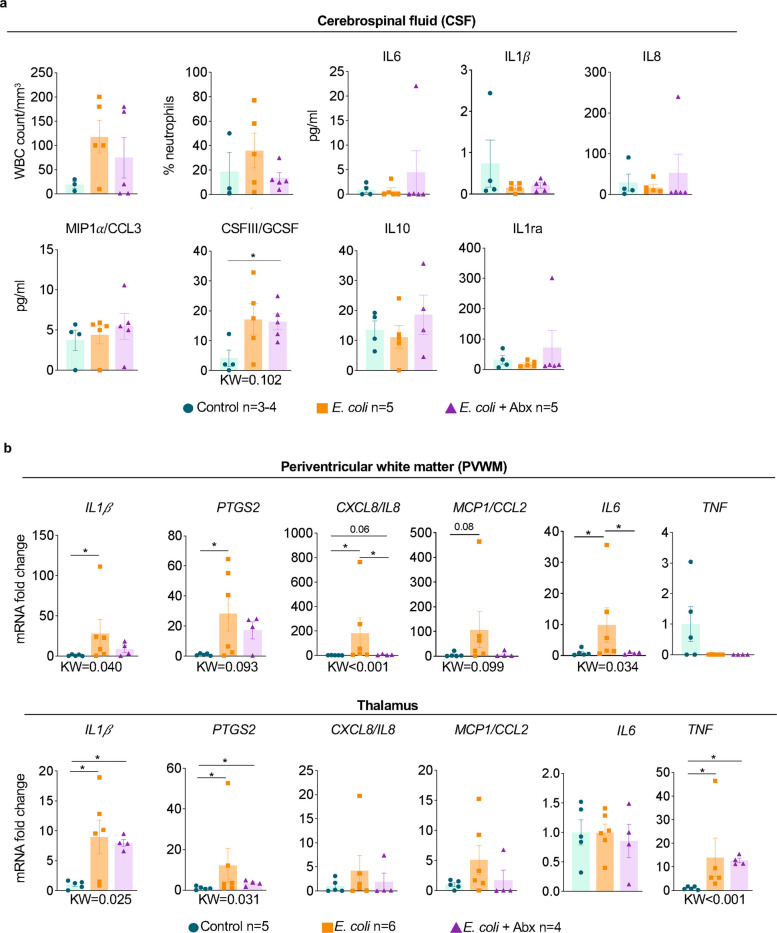
*E. coli* infusion in the chorio-decidua causes neuroinflammation in the fetal periventricular white matter (PVWM) and the thalamus.** a** White blood counts and frequency of neutrophils did not change in the cerebrospinal fluid (CSF) of *E. coli-*exposed animals compared to controls. Overall, pro-inflammatory cytokine levels did not increase upon *E. coli*-exposure. **b** Expression of cytokine mRNAs by qPCR using Taqman probes. In the PVWM there was a significant increase of *IL1β*, *PTGS2, CXCL8/IL8,* and *IL6* in *E. coli*-exposed animals compared to controls. Abx treatment significantly decreased expression of *CXCL8/IL8* and *IL6.* In the thalamus, IL*1β, PTGS2,* and *TNFa* mRNA expression increased significantly upon *E. coli* exposure regardless of Abx treatment compared to controls. Average mRNA values are fold increases over the average value for control after internally normalizing to the housekeeping 18S RNA. Data are mean ± SEM. Initial comparison between three groups were done using Kruskal–Wallis (KW) nonparametric test with p value shown at the bottom of each graph. For two group comparisons, nonparametric Mann–Whitney U test was used (*p < 0.05 between comparators; the actual p values between 0.05 and 0.1 is shown)

Given clinical reports linking FIRS and adverse neurological outcomes, we evaluated inflammation in the central nervous system in our NHP model. Cerebrospinal fluid (CSF) bacterial cultures were negative for all fetuses at delivery (Suppl. Figure 2). CSF white blood counts including neutrophil counts were not elevated in *E. coli* exposed fetuses compared to controls (Fig. 2a). While CSF cytokine levels did not significantly increase in the *E. coli* group, a significant increase in CSFIII/G-CSF was observed specifically in the *E. coli* + Abx group compared to Controls (Fig. 2a).

As a lack of global CSF alterations can mask more circumscribed, focal loci of regional brain inflammation, we probed two key areas for cytokine alterations: the periventricular white matter (PVWM), a region commonly injured in preterm infants and associated with CP pathogenesis [30], and the thalamus, as dysconnectivity in thalamic relay neurons has been implicated in ASD [31]. In the PVWM, compared to sham infection surgical controls, mRNAs for *IL1β, PTGS2 (COX2), CXCL8/IL8,* and *IL6* increased in the *E. coli* group while *MCP1/CCL2* (p = 0.08) and TNF alpha expression were unaltered (Fig. 2b). Antibiotics decreased *E. coli* induced *IL6* and *IL8* levels, but not other cytokine mRNAs in the PVWM. In the thalamus, mRNAs for *IL1β, PTGS2*, and *TNF alpha* increased after *E. coli* exposure with no significant decreases after antibiotics (Fig. 2b). To understand the potential contribution of inflammatory cells in the process, we performed immunostaining in brain tissues for myeloperoxidase (MPO) which is upregulated in neutrophils and monocytes during acute inflammation. *E. coli* exposure increased the number of MPO^+^ cells in fetal brain sections largely located within the blood vessels (Suppl. Figure 3).

### *E. coli* increased microglia and astroglia with reduction in immature neurons in the subventricular zone (SVZ)

To understand the effects of chorioamnionitis/FIRS in the fetal brain, we sought to examine several fetal brain areas critical during development and, by extension, particularly vulnerable in early-life insult models [32, 33]. The areas chosen for detailed neuropathological study are shown (Suppl. Figure 4a). To identify cell types, multi-label confocal microscopy was done using markers for glial, neuronal, and vascular cell types (Suppl. Figure 4b-c).

**Fig. 3:**
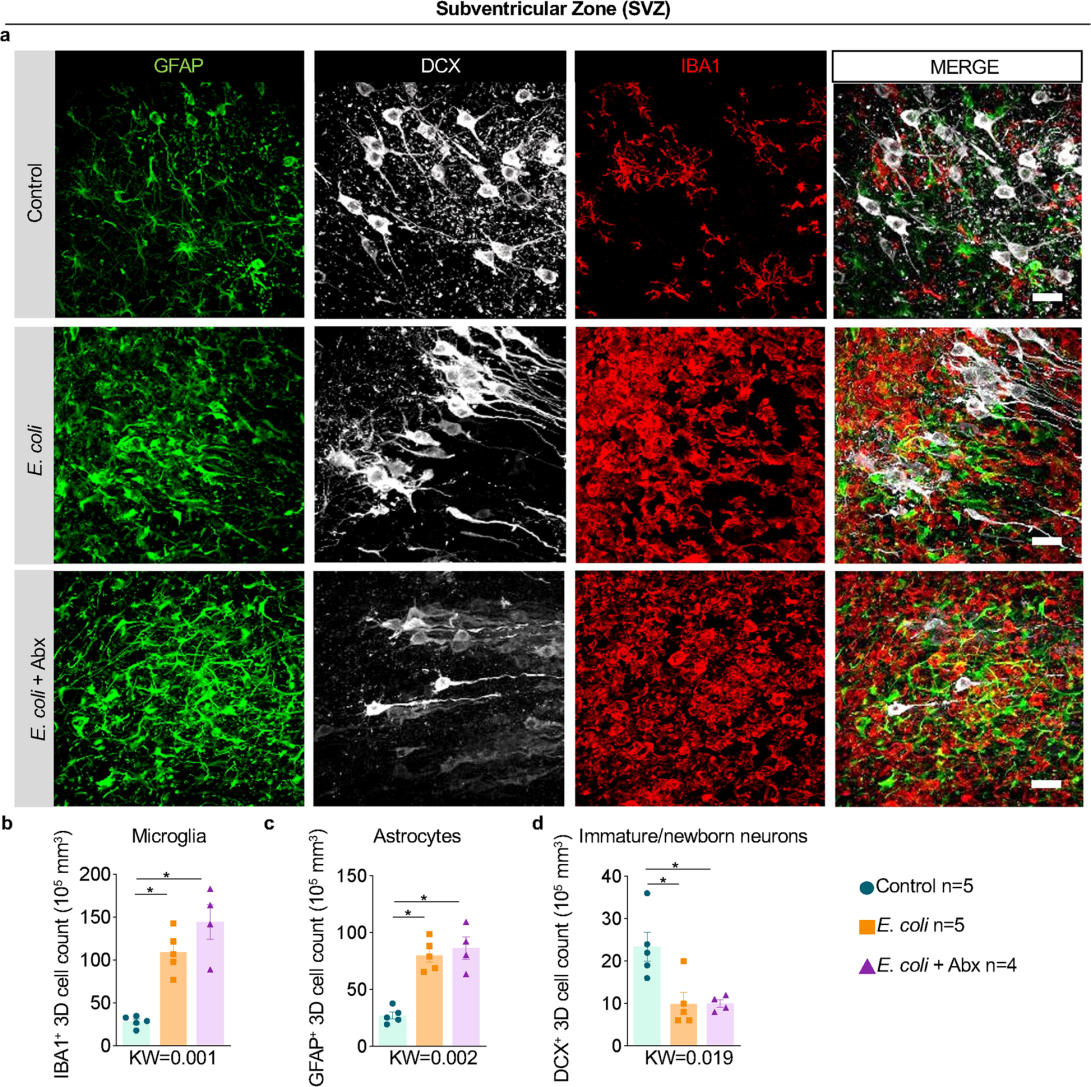
Microglia, astrocytes, and immature neuron changes in fetal subventricular zone (SVZ) by *E. coli* exposure were not improved by maternal antibiotics. Representative images (*n* = 4–6/group) of the SVZ showing multilabel confocal microscopy with the following combinations: **a** newborn/immature neuron marker DCX (white), microglia marker IBA1 (red), and astrocyte marker GFAP (green) across the different experimental groups (Scale bar: 15 µm for panels a and b). Quantitative 3D analysis in the SVZ showed a significant increase in **b** microglial IBA1^+^ population, and **c** GFAP^+^ astrocytes compared to controls regardless of the Abx treatment. **d** There was a significant decrease of DCX^+^ immature neurons upon *E. coli* exposure compared to controls. Data are mean ± SEM. Initial comparison between three groups were done using Kruskal–Wallis (KW) nonparametric test with p value shown at the bottom of each graph. For two group comparisons, nonparametric Mann–Whitney U test was used (*p < 0.05 between comparators)

The SVZ of lateral ventricles adjacent to the periventricular white matter (PVWM) is the site where progenitor niches (both neuron and glia) line up the ventricular space, and is known to be susceptible to perinatal brain injury [24, 32]. Microglia (similar to macrophage) and astrocytes are known to mediate central nervous system inflammatory responses. Following *E. coli* exposure, ionized calcium-binding adaptor molecule-1 (IBA1^+^) microglia and glial fibrillary acidic protein (GFAP^+^) astrocyte cell counts more than doubled in the SVZ of *E. coli* exposed Rhesus fetuses with no effect of antibiotics (Fig. 3a-c). To assess the potential impact of chorioamnionitis/FIRS on neurogenesis, we combined microglial (IBA1) and astrocytic (GFAP) markers with doublecortin (DCX), a microtubule-associated protein that plays an important role in neuronal migration in immature neurons [34]. In contrast to increases in the microglia and astrocytes, DCX^+^ neuron numbers decreased by > 50% in the SVZ (Fig. 3a and d). Critically, neither the reduction in DCX^+^ neurons nor the increase in glial cells was prevented by antibiotic treatment. NeuN, an RNA binding protein [35], is a well-known marker of neurons. Of note, in the SVZ, mature NeuN^+^ neurons were not affected by CD *E. coli* exposure (Suppl. Figure 5). Additional imaging revealed the infiltration of red blood cells (RBC) in the SVZ of *E. coli*-exposed groups with or without Abx, but not in the control group. (Suppl. Figure 6).

### The white matter is vulnerable to neuroinflammation in chorioamnionitis/FIRS

**Fig. 4:**
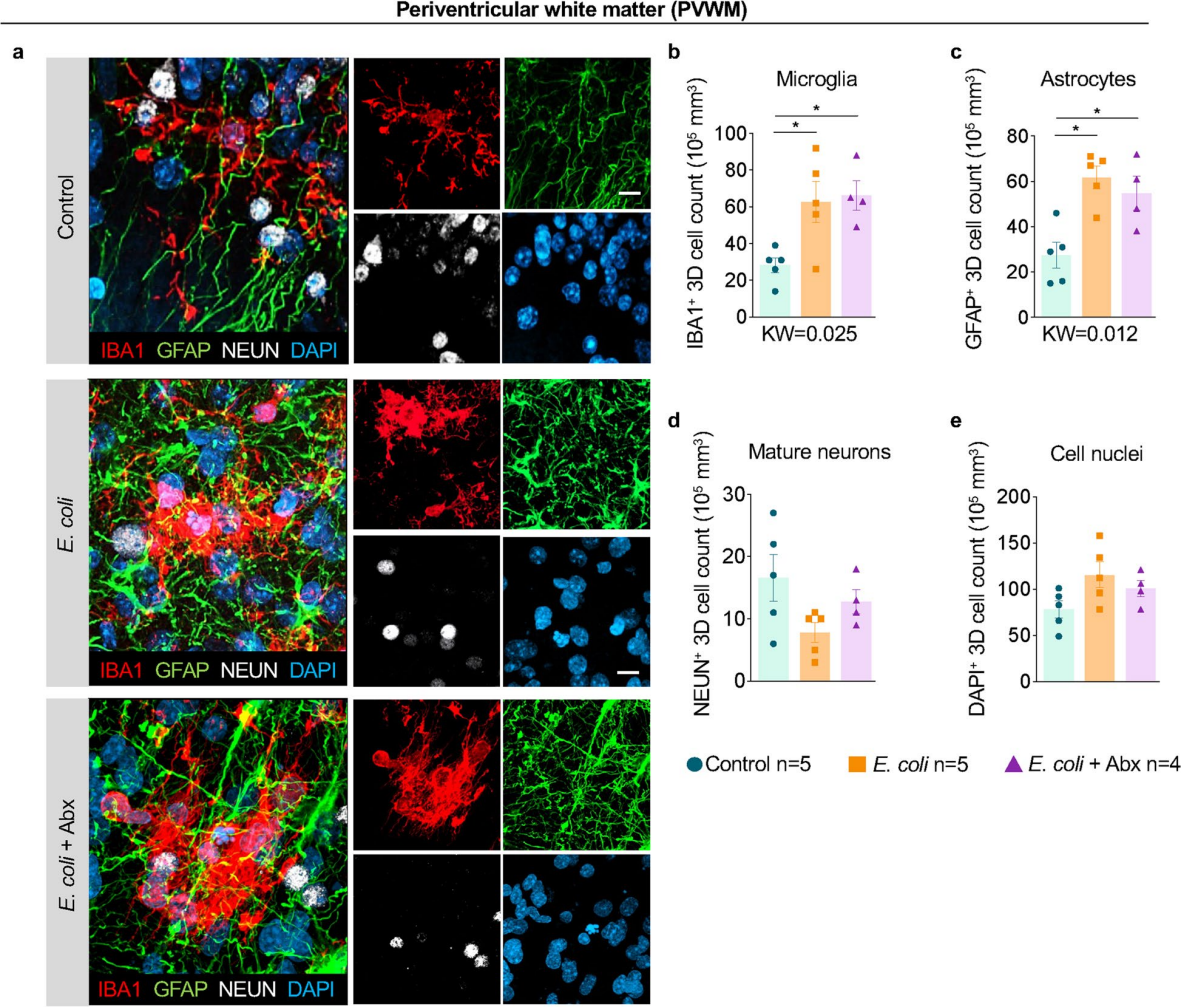
Increased microglia and astrocyte numbers in periventricular white matter (PVWM) by *E. coli* exposure were not reduced by maternal antibiotics. **a** Representative images (*n* = 4–6/group) of the PVWM showing multilabel confocal microscopy combining nuclei marker DAPI (blue), mature neuron marker NeuN (white), microglia marker IBA1 (red), and astrocyte marker GFAP (green) across the different experimental groups (Scale bar: 15 µm). Quantitative 3D analysis in the PVWM showed a significant increase of **b** microglia IBA1^+^ cells, and **c** astrocyte GFAP^+^ cells compared to controls regardless of the Abx treatment. No change in **d** mature NeuN^+^ neurons and in **e** the total number of DAPI.^+^ cells across groups were seen. Data are mean ± SEM. Initial comparison between three groups were done using Kruskal–Wallis (KW) nonparametric test with p value shown at the bottom of each graph. For two group comparisons, nonparametric Mann–Whitney U test was used (*p < 0.05 between comparators)

Following the evidence of elevated pro-inflammatory cytokines in the PVWM (Fig. 2b) and the neuroinflammatory response observed in the nearby SVZ (Fig. 3), we sought to further investigate the cellular response in the white matter of the periventricular and entorhinal areas (Suppl. Figure 4a). In the PVWM, IBA1^+^ microglia and GFAP^+^ astrocyte counts more than doubled after *E. coli* exposure in the PVWM (Fig. 4a). Quantitative 3D analysis of this region confirmed a robust increase in the total microglial population, and astrocytes (Fig. 4b-c), suggesting increased proliferation or translocation of glial cells in the white matter. Maternal antibiotics failed to reverse *E. coli* induced increases in the microglia and astrocyte counts in the PVWM (Fig. 4a-c). There were no significant differences in PVWM NeuN^+^ neuron counts and total cell counts observed between the Control, *E. coli*, and *E. coli* + Abx groups (Fig. 4a, d-e).

**Fig. 5:**
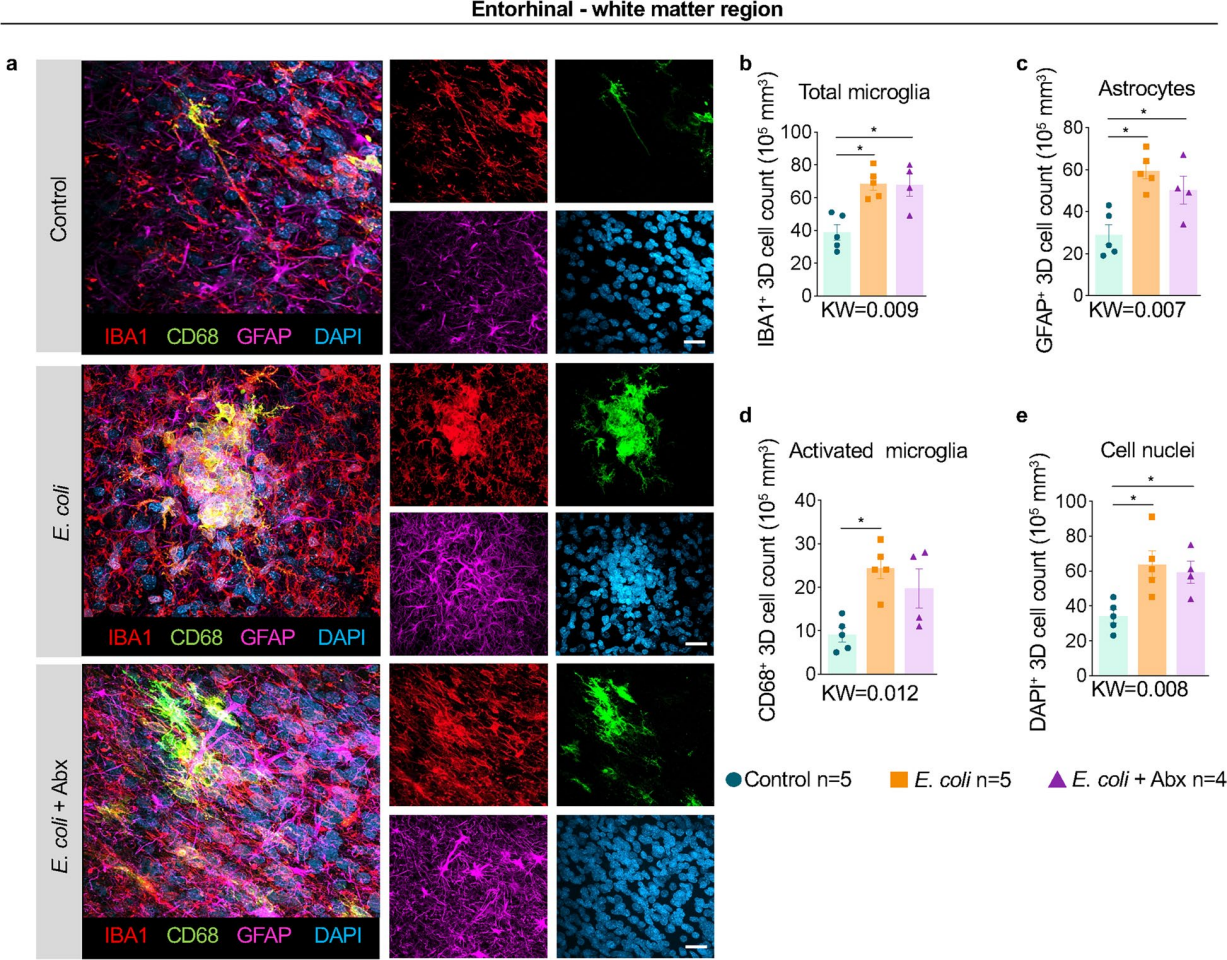
Acute neuroinflammatory response in the entorhinal white matter region of *E. coli* exposed fetuses was not reduced by antibiotics. **a** Representative images (*n* = 4–6/group) showing multilabel confocal microscopy combining nuclei marker DAPI (blue), microglia marker IBA1 (red), microglia activation marker CD68 (green), and astrocyte marker GFAP (magenta) across the different experimental groups (Scale bar: 15 µm). Quantitative 3D analysis in the entorhinal – white matter region showed a significant increase of **b** microglial IBA1^+^ cells and **c** GFAP^+^ astrocytes compared to controls regardless of the Abx treatment.** d** A significant increase in activated microglial CD68^+^ cells in the *E. coli* exposed animals compared to controls animals and **e** DAPI.^+^ cells in the *E. coli* exposed animals were seen compared to controls regardless of the Abx treatment. Data are mean ± SEM. Initial comparison between three groups were done using Kruskal–Wallis (KW) nonparametric test with p value shown at the bottom of each graph. For two group comparisons, nonparametric Mann–Whitney U test was used (*p < 0.05 between comparators)

Similarly, in the entorhinal white matter, we observed clusters of reactive microglia and astrocytes, particularly in the deeper white matter regions (Fig. 5a). This region in the medial temporal lobe contains fibers that connect the hippocampus and neocortex and is implicated in ASD due to its role in memory and spatial processing [36]. *E. coli* exposure regardless of maternal antibiotics increased IBA1^+^ microglia and GFAP^+^ astrocyte counts in the entorhinal white matter region (Fig. 5a-c). These microglia often expressed CD68 indicating activation and increased phagocytic activity and were accompanied by hypertrophied astrocytic process (Fig. 5a). Quantification of 3D images confirmed a significant rise in both activated microglial and astrocytic populations driving increased cell numbers in this region following *E. coli*. Notably, these changes were again demonstrated to be resistant to maternal antibiotic treatment (Fig. 5 b-e).

### Selective impairment of immature neurons in the dorsolateral prefrontal cortex

**Fig. 6:**
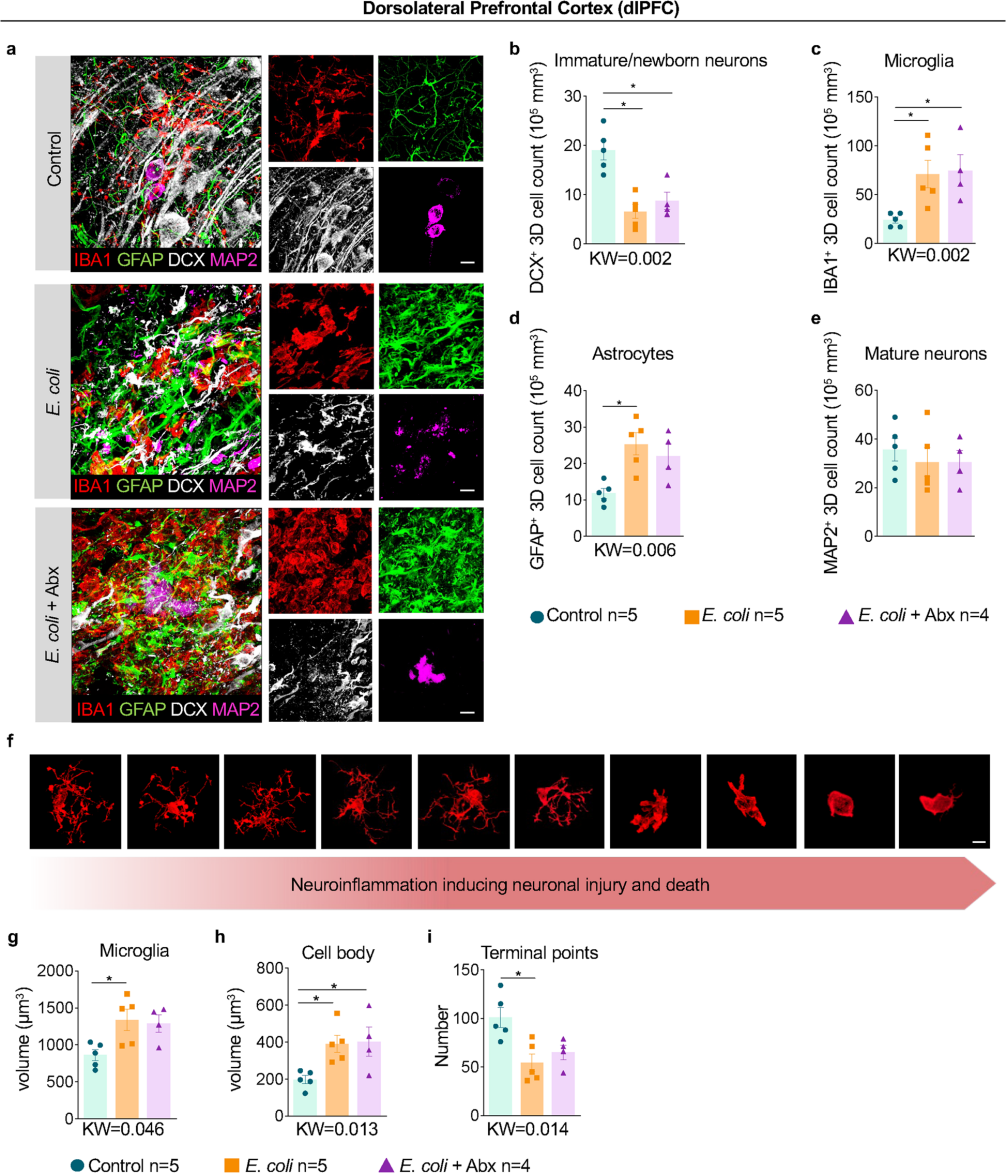
Selective decrease in the immature DCX^+^ neurons but not mature MAP2^+^ neurons in the dorsolateral prefrontal cortex (dlPFC) by *E. coli* is not reversed by maternal antibiotics. Representative images (*n* = 4–5/group) of the dlPFC showing multilabel confocal microscopy combining **a** mature neuron marker MAP2 (magenta), immature neuron marker DCX (white), microglia marker IBA1 (red), and astrocyte marker GFAP (green) across the different experimental groups (Scale bar: 15 µm). Quantitative 3D analysis in the dlPFC showed a significant decrease of **b** total DCX^+^ immature neurons, a significant increase in **c** IBA1^+^ microglia cells, **d** GFAP^+^ astrocytes, and no change in **e** MAP2^+^ mature neuron numbers across the groups compared to controls regardless of the Abx treatment. **f** Illustrative photomicrographs showing the spectrum of changes that microglia undergo, from highly ramified, small volume microglia (left, low activation), to ameboid, highly activated microglia with few or no processes (right). A significant **g** increase in total volume of microglia, **h** enlargement of the cell body, and **i** a significant decrease in the number of microglial processes (i.e., total number of terminal points) was observed after *E. coli* exposure regardless of antibiotics compared to control animals (Scale bar: 5 µm). Amoeboid shape and retracted processes of microglia are characteristic of activation. Data are mean ± SEM. Initial comparison between three groups were done using Kruskal–Wallis (KW) nonparametric test with p value shown at the bottom of each graph. For two group comparisons, nonparametric Mann–Whitney U test was used (*p < 0.05 between comparators)

We next examined the neuronal population of the dorsolateral prefrontal cortex (dlPFC) (Suppl. Figure 4a), an area critical for high-order cognitive processing in humans that develops late in the gestational period and is implicated in ASD and intellectual disabilities. In the dlPFC, exposure to *E. coli* resulted in a selective reduction in the DCX^+^ neuronal population, suggesting an overall reduction in migrating neurons to this area (Fig. 6a). Quantification of 3D stacks confirmed this sharp reduction in the DCX^+^ population (Fig. 6b), as well as robust increases in microglia (IBA1^+^) and astrocytes (GFAP^+^) following *E. coli* exposure (Fig. 6c-d). Critically, antibiotic treatment did not reverse the decrease in DCX^+^ cells or the increase in microglia in the dlPFC, while having a mixed effect on the astrocytic population (Fig. 6b-d). Interestingly, no significant changes were observed in the mature MAP2^+^ neuronal population (Fig. 6e), indicating that immature rather than mature neurons are particularly vulnerable to chorioamnionitis/FIRS-related insult. To further examine whether dlPFC alterations were potentially mediated by neuroinflammatory changes, we investigated microglial activation using well-established morphological parameters. These include morphometric measurements of microglial volume, soma volume, and terminal points. Notably, more activated microglia exhibited an ameboid shape, known to be a reliable marker of increased phagocytic activity (Fig. 6f) [37]. Importantly, compared to controls, microglia in the dlPFC of *E. coli* exposed fetuses regardless of maternal antibiotics have increased total and somatic volume, as well as a reduction in terminal points, consistent with microglial activation (Fig. 6g-i).

Given the important role of neurogenesis in the formation and maturation of the hippocampal formation (HF) and the significant neuroinflammation observed in the adjacent entorhinal white matter, we investigated the inferior temporal lobe for both inflammatory and neurogenic changes. In contrast with the white matter regions (Fig. 5a-c), no substantial changes in neuroinflammation were present in the entorhinal cortex (EC), and the number of mature, NeuN^+^ neurons was stable between groups (Suppl. Figure 7a-e). In the HF, we observed a qualitative increase in microglia volume and density (Suppl. Figure 8a-c), but no effect in the incorporation of mature MAP2^+^ neurons in the dentate gyrus (Suppl. Figure 9).

## Discussion

**Fig. 7:**
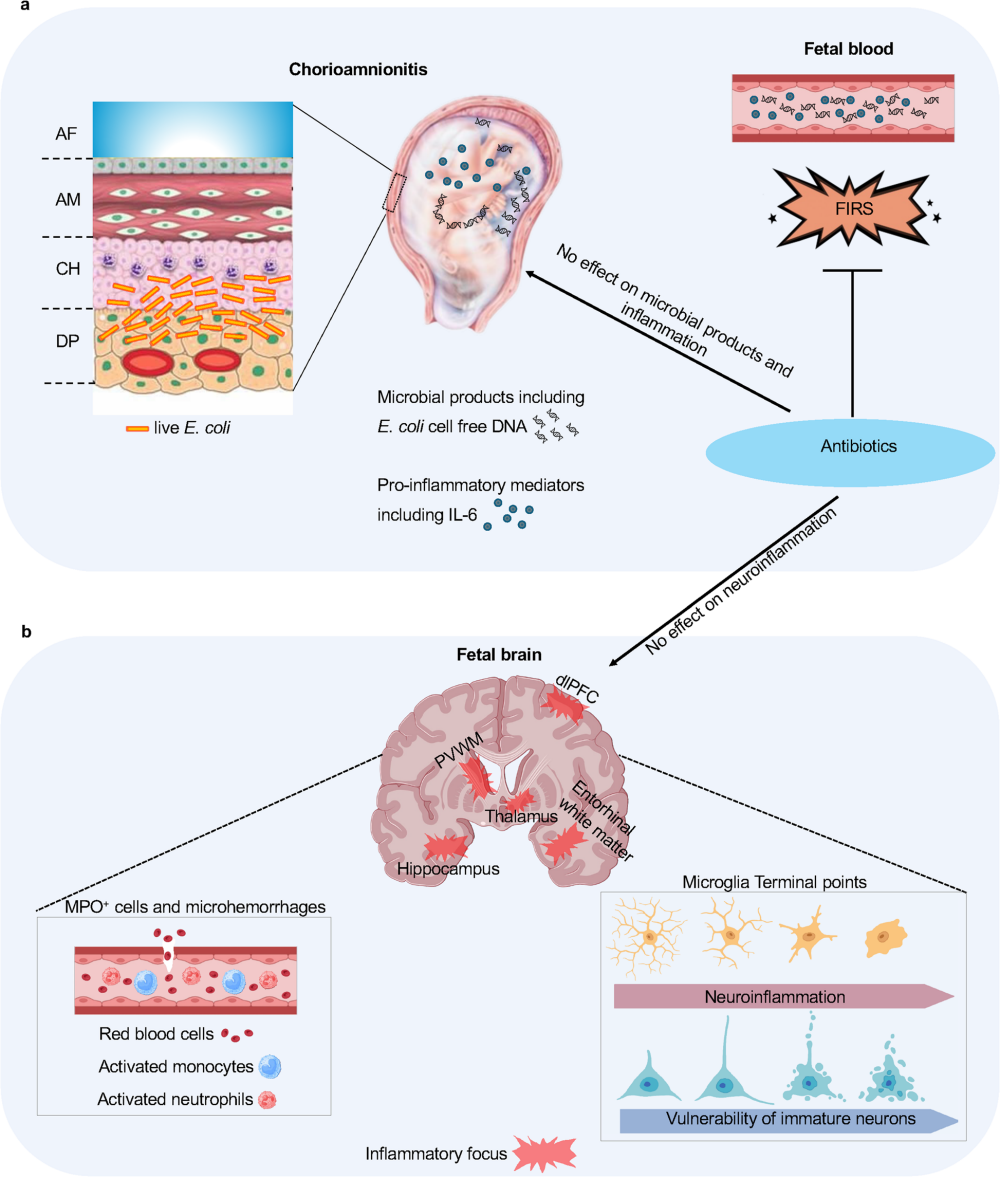
Model of fetal neuroinflammation induced by localized infection in the chorio-decidua.** a** Chorioamnionitis most often causes a localized chorio-decidua infection. Immune system and/or maternal antibiotics can kill microorganisms, but bacterial products including cell-free DNA diffuse in the amniotic fluid and the cord blood. The resulting inflammatory response in the amniotic fluid is not significantly decreased by maternal antibiotics. Inflammatory response in the cord blood (termed fetal inflammatory response syndrome or FIRS) is inhibited by maternal antibiotics. AF = amniotic fluid; AM = amnion; CH = chorion; DP = decidua parietalis. **b** Bacterial products in the fetal circulation induces neuroinflammation through microglia activation, astroglia activation, activated MPO^+^ neutrophils/monocytes and microhemorrhages. The resulting neuronal injury is characterized by a vulnerability of DCX^+^ immature/newborn neurons particularly in white matter regions without a significant impact on mature cortical neurons. Importantly, antibiotic treatment is not effective in decreasing neuroinflammation or neuronal injury

In this study, we describe a new model of chorioamnionitis in the non-human primate evaluating acute fetal brain inflammatory responses after a 72 h exposure. This model addresses a critical well-documented conundrum that chorioamnionitis induced by lower genital organisms are rarely detected in the fetal blood even in the face of significant systemic fetal inflammation (Fig. 7a). In our study, the cytokine profiles observed in cord blood were strikingly similar to clinical reports of infants with FIRS-induced neurological impairments [4, 14–16] further corroborating the goal of the model to emulate chorioamnionitis/FIRS. We also show that chorioamnionitis/fetal inflammatory response syndrome (FIRS) induced a robust fetal neuroinflammatory response characterized by microglial and astrocytic activation, and neurovascular alterations. Additionally, there was a selective reduction in the immature neurons expressing DCX, with no significant impact on mature neuronal populations expressing MAP2 or NeuN (Fig. 7b). The white matter areas particularly the peri-ventricular white matter and the entorhinal white matter were most susceptible to injury. Fetal neuroinflammation occurred despite the absence of bacteremia or bacterial invasion in the central nervous system, suggesting that microbial products (pattern-associated molecular pattern, PAMPS) are likely drivers of the fetal inflammatory response. These products may reach the fetus directly via the umbilical cord or by pulmonary aspiration of the amniotic fluid [12]. Critically, antibiotic treatment reduced the severity of FIRS but did not attenuate neuroinflammation or neuropathology. A major implication of our study is that adjunctive immunomodulatory therapies need to be investigated in treating chorioamnionitis/FIRS-associated neurodevelopmental impairments. Overall, the study provides mechanistic insights into the strong epidemiological associations between chorioamnionitis/FIRS and adverse child neurological outcomes (Fig. 7).

Since direct microbial invasion in the central nervous system was ruled out, *E. coli*-derived products in the fetal circulation likely initiated the inflammatory response. While endotoxin is a potential mediator, antibiotics reduced endotoxin activity in cord blood (Fig. 1d) without decreasing neuroinflammation, suggesting that endotoxin is less likely to be involved. On the other hand, previous studies have demonstrated that both hypoxia–ischemia and endotoxin can cause brain injury [38, 39]. Notably, antibiotics did not reduce high levels of *E. coli* cell-free DNA in the amniotic fluid (Fig. 1c). Indeed, microbial cell-free DNA is known to persist for a much longer duration than live bacteria after antibiotic therapy [28]. Despite the ubiquitous presence of DNAses, microbial cell-free DNA can persist possibly due to sequestration within biofilms, exosome vesicles, or plasma proteins. Alternatively, and non-exclusively, it can come from the continuous release of DNA fragments by necrotic cells or other sources [40]. Our model of continuous infusion of *E. coli* in CD space models the prolonged release of bacterial products from hidden niches. Microbial cell-free DNA is a potential mediator of neuroinflammation, since innate immune cells, including microglia, can react to DNA sensing through different pathways, including TLR9 sensing hypomethylated CpG microbial DNA motifs, cGAS-STING, and AIM2 pathways [29], leading to the release of pro-inflammatory cytokines, particularly IL1β and Type I interferons. In support of this hypothesis, we saw that *E. coli* increased *IL1β* mRNA in both the PVWM and the thalamus.

The regional specificity of the inflammatory response is particularly striking. Microglial and astrocytic activation was robust in the PVWM and entorhinal white matter, areas known to be particularly vulnerable to inflammatory damage. Although this damage is more prominent in the preterm, term infants are also susceptible to chorioamnionitis induced brain injury [7]. Microglia activation can directly cause neurotoxicity via metalloproteinases and reactive oxygen or nitrogen species, or indirectly via the release of pro-inflammatory cytokines and glutamate, leading to excitotoxic injury [41, 42]. Reactive astrocytes enveloping the blood vessels can reciprocally signal with activated microglia thereby causing neurovascular alterations contributing to brain inflammation [43]. In contrast to white matter regions, cortical layers had less severe pathology. This differential vulnerability supports the idea that specific white matter regions are more prone to fetal inflammatory responses, potentially due to their higher concentration of activated microglia. This distinctive pattern of injury observed in our model (focal, non-cystic white matter lesions) resembles patterns commonly observed in premature infants [44]. While these injuries are classically reported in the context of hypoxia, ischemia, or hypotension, our results uniquely demonstrate that chorioamnionitis/FIRS without CSF pleocytosis can induce this characteristic pattern of injury.

Another important discovery of this study was the vulnerability of migrating DCX^+^ immature cell populations, compared to the relative resilience of mature MAP^+^ and NeuN^+^ neuronal populations in cortical areas. These observations are consistent with the reported susceptibility of immature neurons to stress-induced cell death, which may be attributed to an increased vulnerability to excitotoxicity [44, 45]. Critically, by acting selectively on immature neurons, the timing of the infection becomes a major determinant of the neurodevelopmental consequences of FIRS, which should be considered in both clinical practice and when trying to develop clinical and pharmacological interventions. In primates, neurogenesis and gliogenesis in the SVZ continue into the third trimester, playing a crucial role in producing neurons that integrate into the cortical mantle circuitry in an inside-out fashion [25, 32, 33, 46]. Our observation of increased microglia and astrocytes in the SVZ, accompanied by a decrease in DCX^+^ neurons, supports this area as a potential vulnerable region during chorioamnionitis/FIRS.

Our neuropathological observations have significant clinical importance. Intraventricular hemorrhage, a common complication of prematurity, is known to be a major cause of PVWM injury, a well-known risk factor for cerebral palsy, behavior disorders, cognitive delays, and neurosensory impairments in childhood [30, 47]. A mouse model overexpressing VEGF in the germinal matrix induced intraventricular hemorrhage in the late embryonic period via increased neurovascular proteases [48]. Systemic inflammation induced by LPS caused cerebral microhemorrhages via endothelial activation, blood–brain barrier disruption, and neuroinflammation in adult mice [49]. Notably, systemic inflammation has also been associated with cerebral microhemorrhages in the elderly human population [50]. Also, systemic inflammation has been reported to increase diffusion of pro-inflammatory mediators into the central nervous system even without anatomical disruption of the blood brain barrier [51]. Consistently, we observed a modest infiltration of myeloperoxidase (MPO) positive cells in the brain vessels of *E. coli* exposed fetuses. Another possibility is that activated microglia and other immune cells in the perivascular space could have contributed to neurovascular alterations as has been reported in the immature brain [52]. Collectively, MPO^+^ cells and cerebral microhemorrhages could be potential contributors to the neuroinflammation in our study.

Furthermore, neuroinflammation and loss of immature neurons in the dlPFC, thalamus, and hippocampus would be consistent with epidemiological reports of increased susceptibility to brain injury in the neonatal period, as well as associations between ASD, neurobehavioral disorder, cognitive delays, and executive dysfunction in infants exposed to intrauterine infections [6, 7, 53].

Perhaps an even more clinically relevant observation is that antibiotics given after exposure to the infectious agent can improve fetal inflammation but have limited efficacy in reversing neuroinflammatory and neurodevelopmental consequences. While antibiotics are the current standard treatment for chorioamnionitis/FIRS [54, 55], our results suggest that fetal protection is not complete. Although our study was not designed to evaluate the potential deleterious effects of maternal antibiotics, large epidemiological studies have suggested that maternal antibiotics may even exacerbate the risk for ASD due to possible disruption of developing gut microbiome [23]. Taken together, our data support the concept that anti-inflammatory therapies might have a neuroprotective effect in chorioamnionitis/FIRS. IL1 receptor antagonist (IL1ra, Anakinra) and anti TNF⍺ antibody (Adalimumab) have shown efficacy in decreasing chorioamnionitis induced maternal/fetal inflammation and brain injury in animal models [47, 56–58]. Furthermore, maternally given Anakinra reaches therapeutic concentrations in the fetus of Rhesus macaques [56], highlighting the potential of NHP models to test candidate interventions, including the most effective combination of prenatal antibiotics and anti-inflammatory therapeutics.

The study is not without its limitations. The rationale for the 3rd trimester infectious exposure was to model intrauterine infection. However, neuro-imaging studies reveal that Rhesus macaque brain at 85% gestation is more similar to 42-week gestation human brain [26]. Thus, brain regional vulnerability observed in this study may be more representative of term rather than preterm human brain. Interestingly, both preterm and term infants are susceptible to chorioamnionitis induced brain injury. While we did not observe differences in brain inflammatory markers or FIRS measurements between animals with and without preterm labor within each group, we acknowledge that the study was not powered to detect subtle effects. Therefore, we cannot definitively exclude the possibility that preterm labor contributed to some aspects of the observed outcomes in a subset of animals. Indeed, preterm labor and neuroinflammation were concurrently observed in animal models of inflammation induced preterm birth [59]. Neuroinflammatory responses to FIRS caused by a virulent microorganism such as *E. coli* are likely different from less virulent organisms that most frequently cause chorioamnionitis in the Western world, such as *Ureaplasma* species [3]. Furthermore, the use of a single infectious agent is not sufficient to capture the variety of clinical presentations of FIRS seen in developing and developed nations [60, 61]. This study is also focused on a relatively short period of infection and treatment (72 h), which is insufficient to fully characterize residual inflammatory responses and their consequences in childhood neurological conditions. Despite these considerations, the study highlights the possibilities for more in-depth mechanistic studies using in vivo and ex vivo approaches that leverage this FIRS model. As an example, future studies could employ organ-on-chip or organoid approaches to more precisely understand the mechanisms.

In summary, the NHP model described in this study demonstrates that localized chorio-decidua infection with *E. coli* results in FIRS without bacteremia, leading to significant neuroinflammation driven by microglia and astroglia, particularly in white matter regions. Selective vulnerability of developing neurons was also observed. These findings after 72 h exposure to microbial products provide insights into the pathogenesis of FIRS and suggest that adjunctive anti-inflammatory therapies, in addition to antibiotics, may be needed to mitigate the adverse neurodevelopmental effects associated with this condition.

## Methods

### Animals and *E. coli* infusion

Normally cycling adult female Rhesus macaques (*Macaca mulatta*) (*n* = 19) were time mated. At ∼140 days of gestation (∼85% of term gestation), a micro-osmotic pump (Alzet, model 2001; 1.0 μl/hr) loaded with 1 ml of sterile LB broth or live *E. coli* in LB broth was surgically implanted in the chorio-decidua of pregnant Rhesus macaques via mini-laparotomy and hysterotomy. Three groups of animals were randomly allocated infusions as follows: 1) Controls, *n* = 7 infusing diluted sterile LB broth; 2) Live *E. coli n* = 7; 3) Live *E. coli* + maternal antibiotics (Abx) (*n* = 5) (Suppl. Table 1 and Fig. 1a). The strain of *E. coli* UTI189 used for experiments was from a patient with urinary tract infection. Frozen UTI89 vials were washed and resuspended in 50% saline:50% LB broth medium. *E. coli* infusion was calibrated to infuse at 10^5^ CFU/h for 3 d (total 7 × 10^6^ CFU). A catheter with dead space volume of 24µL was attached to the pump for all the groups to allow LB broth/*E. coli* infusion for 24 h after implantation (Fig. 1a). Although the 24 h time prior to beginning continuous infusion was designed to allow for a rest period, some leakage of infusate cannot be excluded. In group 3, antibiotics (Cefazolin 25 mg/k + Enrofloxacin 5 mg/k; twice daily) were given for 3 d via maternal intramuscular route. Antibiotics were started 48 h after the pump implantation or 24 h after the start of *E. coli* infusion (Fig. 1a). Fetuses were delivered surgically 4 days after pump implantation (72 h after E. *coli*/broth infusion), euthanized with pentobarbital, and fetal tissues/fluids were collected. Fetal cerebrospinal fluid (CSF) was collected carefully without causing blood vessel trauma by tapping the foramen magnum.

### Histologic evaluation of fetal membranes for chorioamnionitis

H&E-staining was done on fixed paraffin embedded sections of fetal membranes and scored in a blinded manner for chorioamnionitis (SGK) using well established criteria [62]. This scoring is based on numbers and tissue plane of neutrophil infiltration (Suppl. Figure 1).

### Fetal brain tissue preparation

Fetal brains were rapidly harvested during necropsy, hemisected, and right brain was used for immunohistology while the left brain was further dissected, and different regions were snap frozen for RNA extraction. The right hemisphere, with intact meninges, was subdivided into four anteroposterior blocks and fixed by immersion in 4% formaldehyde with 0.125% glutaraldehyde in phosphate buffer (pH 7.4) at 4 °C for 48 h. After buffer rinses, brain blocks were stored in phosphate-buffered saline (PBS) with 0.1% sodium azide until further processing. Brain blocks containing the dlPFC, EC, HF, and PVWM were then sectioned into semi seriated, 50 μm-thick coronal sections in a vibratome. Sections were stored in PBS with 0.1% sodium azide at 4 °C.

### *E. coli* culture and cell free DNA* E. coli* detection

Maternal/umbilical cord blood, AF, CSF, chorio-decidua, and liver tissue lysate were inoculated in blood culture vials and transported to microbiology laboratory where a defined volume of inoculum was plated on LB agar using loops. Colony growth was reported semi quantitatively after 48 h of incubation (scale 0 to 4 +). Microbial cell-free DNA (mcfDNA) was processed in the amniotic fluid and sequenced as previously described [63] using the RUO version of the Karius assay with amniotic fluid as the input analyte. Sequencing reads were interpreted with the Karius bioinformatic pipeline (version DC-4.1), which maps mcfDNA to 16,000 distinct species. For differential-abundance analysis, we applied the Mann–Whitney U test to each predefined sample group. The mcfDNA assay could not be run on cord plasma since this assay is only validated for EDTA anti-coagulant, and all our cord blood samples were stored using heparin anti-coagulant.

### Endotoxin activity assay

Endotoxin level in the cord plasma was determined by Limulus amebocyte lysate assay (LAL; Lonza) according to manufacturer’s instructions.

### Cytokines, Prostaglandins, and C-reactive protein (CRP) ELISA

Cytokine/chemokine concentrations in AF, cord plasma, and CSF were determined by Luminex using non-human primate multiplex kits (EMD Millipore, cat#PRCYOTMAG-40 K). Lipids were extracted from the AF using methanol to measure prostaglandins PGE2 and PGF2_a_ (Cayman Chemical, cat#514,010 and cat#516,011). C-reactive protein (CRP) concentration was measured in cord plasma at delivery (CRP, Life Diagnostics, cat#CRP-3).

### RNA extraction and quantitative PCR (qPCR)

Total RNA was extracted from snap-frozen PVWM and thalamus with RNEasy Mini kit (Qiagen), according to manufacturer’s instructions. RNA concentration and quality were measured by Nanodrop spectrophotometer (ThermoScientific). Reverse transcription of the RNA and quantitative RT-PCR were performed using qScript One-Step RT-qPCR Kit (Quanta Biosciences), following the manufacturer’s instructions and with Rhesus-specific TaqMan gene expression primers (Life Technologies) (Suppl. Table 2). Eukaryotic 18S rRNA (Life Technologies) was endogenous control for normalization of the target RNAs.

### Immunostaining and cell counting

Multi-parameter immunofluorescence was performed on brain sections as described [64]. Briefly, antigen retrieval was performed by incubating free-floating brain sections with citrate buffer pH 6.0 (S1700; FUJIFILM Wako, Osaka, Japan) at 60^◦^C for 30 min. After washes in PBS, off-target antibody binding blocking and membrane permeabilization were performed by incubating sections with a 5% normal donkey serum, 5% normal goat serum, and 5% bovine serum albumin solution in PBS with 0.3% triton for 2 h at room temperature under agitation. Sections were then incubated for multiplex immunohistology: NeuN (1:500), MAP2 (1:1,000), DCX (1:1,000), IBA1 (1:400), GFAP (1:1,000), CD68 (1:200) (see Suppl. Figure 4 for antibody details). Following primary antibody incubation, sections were incubated with the respective fluorophore-conjugated secondary antibodies (AlexaFluor 405, 488, 555, and 647; Invitrogen) for an additional 48 h at 4 °C. When appropriate, sections were stained with DAPI (1:5,000) for 15 min at room temperature to label nuclei. Finally, sections were mounted onto microscopy glass slides and cover slipped using ProLong Gold (Invitrogen) to minimize fading and photobleaching. Immunolabeled sections were imaged in an AxioImager Z2 microscope (Zeiss) equipped with four laser lines (405 nm, 488 nm, 561 nm, and 640 nm) and a confocal head (LSM800) equipped with two GaAsP PMT detectors and a super-resolution detector array (Zeiss Airyscan). Target brain areas were identified using a combination of DAPI and cell-specific immunolabeling based on the parcellation as defined at lower magnification [65]. For quantification, multiple Z-stacks were acquired using Zeiss’ proprietary software ZEN 2.4 for each region using either a 20X or 63X objective. All acquired images were saved unretouched, including all metadata. For morphometric analysis,.czi files were exported from ZEN and imported into the Imaris 9.8 software suite (Oxford Instruments) for guided, semi-automated image segmentation. Briefly, an experimenter blind to experimental groups defined threshold and filter criteria to generate the most accurate 3D/segmentation of the image for each channel, taking into account labeling strength, autofluorescence, and potential artifacts. Morphometric data were extracted from the segmented images, including the number of objects, volume, ramification, soma roundness, and colocalization. We have extensively employed this analytical approach for histological data with demonstrated data consistency [66, 67].

For MPO staining, a rabbit polyclonal MPO ab (abcam, dilution at 1:50) was used. All 4 µm paraffine sections were subjected to a heat antigen retrieval step before applying the first antibody. Slides were treated with AR-10 (Biogenex) for 2 min at 125 °C in the Digital Decloaking Chamber (Biocare), followed by cooling to 90 °C for 10 min. The EnVision systems (Agilent) were used as the detection system, with AEC (Agilent) as a chromogen. The slides were counterstained with Gill’s hematoxylin I (StatLab). Primary antibody was replaced by rabbit isotype control (ThermoFisher) and ran with each staining series as the negative control. Slides were visualized and Images were captured with Zeiss Imager Z1 (Carl Zeiss).

### Statistical analyses

GraphPad Prism software (version 9.0) was used to graph and analyze statistical significance. Values were expressed as means ± SEM. For non-normally distributed data, initial three group comparisons were done using the Kruskal–Wallis test for non-parametric data. To further understand two group comparisons, a two-tailed Mann–Whitney *U* test for non-parametric data was used. For Gaussian distributed data, paired t test was used. Results were considered significant for *p* ≤ 0.05. Details tests are reported in each figure legend.

## Supplementary Information

Supplementary Material 1.

## Data Availability

All data generated or analyzed during this study are included in this published article [and its supplementary information files].
