# Supplementary material for: Diffuse neuroinflammation and immature neuron loss in fetal Rhesus macaques after short-term intrauterine infection
Source: J Neuroinflammation. 2026 Jan 15;23:60. doi: 10.1186/s12974-025-03686-y (PMC12892564; doi:10.1186/s12974-025-03686-y)
Supplement: Supplementary file 1 — Supplementary Material 1. [file 12974_2025_3686_MOESM1_ESM.docx]

**Diffuse neuroinflammation and immature neuron loss in fetal Rhesus macaques after short-term intrauterine infection**

**Presicce et al.** Manuscript ID: 58232b8b-bdac-4af8-b720-e43385c56938

**Supplementary Figure 1. *E. coli* infusion in the chorio-decidua space induces chorioamnionitis.** Representative (n=4-5/group) fetal membrane (chorioamnion-decidua) H&E histology. Note that magnification showed neutrophil infiltration in the chorio-decidua, a hallmark of chorioamnionitis. a = amnion; c = chorion; d = decidua parietalis.

**Supplementary Figure 2. *E. coli* growth was not observed in the cerebro-spinal fluid (CSF).** *E. coli* gwoth in the fetal CSF was evaluated using traditional culture methods at delivery. No growth of *E. coli* was seen in any animal.

**Supplementary Figure 3. *E. coli* infusion in the chorio-decidua space induces MPO^+^ cell infiltration in the fetal brain.** Representative (n=2-3/group) myeloperoxidase (MPO) staining in the fetal brain. Green arrows indicate MPO^+^ cells. *E. coli* infusion in the chorio-decidua increase the number of MPO^+^ cells.

**Supplementary Figure 4. Information regarding brain regions, cell types and antibodies used in this study. a** At delivery, fetal brains were collected, and different regions were used in the study for multi-parameter 3D confocal immunohistology. **b** Cell types and markers investigated in the study. **c** Antibodies used in the immunohistology analysis.

**Supplementary Figure 5.** No changes in mature neurons upon *E. coli*-exposure in fetal subventricular zone (SVZ). Representative images (n=4-6/group) of the SVZ showing multilabel confocal microscopy with the following combinations: nucleus marker DAPI (blue), microglia marker IBA1 (red), astrocyte marker GFAP (green), and mature neuron marker NeuN (white) across the different experimental groups.

**Supplementary Figure 6. CD *E. coli* exposure results in red blood cell (RBC) infiltration of the periventricular space and is not reduced with maternal antibiotic treatment.**  Representative images (n=4-5/group) of the subventricular zone (SVZ) showing multilabel confocal microscopy combining the immature neuronal marker DCX (purple), mature neuronal marker MAP2 (white), astrocytic marker GFAP (green), microglial marker IBA1 (blue), and spectral labeling of red blood cells (RBC - red). No RBCs were found in the SVZ of control animals. Numerous RBCs were observed in the SVZ of animals exposed to *E. coli*. RBCs persist in the SVZ despite maternal antibiotic treatment. (Scale bar: 5 µm).

**Supplementary Figure 7. No inflammatory response or reduction in mature neuronal population was observed in the entorhinal cortex (EC) following CD *E. coli* exposure. a** Representative image (n=4-5/group) of the EC showing multilabel confocal microscopy combining the nucleic marker DAPI (blue), neuronal marker NeuN (white), microglial marker IBA1 (red), and astrocytic marker GFAP (green). **b-e** Quantitative 3D analysis of the EC shows no significant alterations in the gray matter across multiple parameters, including the number of microglia, astrocytes, neurons, and nuclei. Scale bar: 20 µm.

**Supplementary Figure 8. Increased IBA1^+^ immunosignal in the hippocampal formation of *E. coli*-exposed animals.** Representative images (n=4-5/group) of the hippocampal formation (HF) showing multilabel confocal microscopy combining the nucleic marker DAPI (blue) and the microglial marker IBA1 (red). **(a, a’)** In control animals, microglia are highly ramified and have low somatic volumes. **(b, b’)** In *E. coli*-exposed animals, there is a marked increase in IBA1 immunosignal, with higher magnification photomicrographs revealing enlarged soma volumes for microglia in the area. **(c, c’)** In Abx-treated animals, microglia display a morphology similar to that of untreated animals, suggesting that Abx treatment does not reverse hippocampal neuroinflammation. (Scale bar: A-C 250 µm; A’-C’ 50 µm).

**Supplementary Figure 9. *E. coli* infusion does not affect incorporation of mature neurons in the hippocampus, despite microglia activation.** Representative images (n=4-5/group) of the hippocampal formation showing multilabel confocal microscopy combining the nucleic marker DAPI (blue), mature neuron marker MAP2 (white), microglial marker IBA1 (red), and astrocytic marker GFAP (green). No significant MAP2 immunolabeling alterations were observed among groups, despite increases in microglial and astrocytic immunolabeling between controls and *E. coli*-exposed groups. (Scale bar: 40 µm).
